# Downregulation of *de Novo* Fatty Acid Synthesis in Subcutaneous Adipose Tissue of Moderately Obese Women

**DOI:** 10.3390/ijms161226206

**Published:** 2015-12-16

**Authors:** Esther Guiu-Jurado, Teresa Auguet, Alba Berlanga, Gemma Aragonès, Carmen Aguilar, Fàtima Sabench, Sandra Armengol, José Antonio Porras, Andreu Martí, Rosa Jorba, Mercè Hernández, Daniel del Castillo, Cristóbal Richart

**Affiliations:** 1 Grup de Recerca GEMMAIR (AGAUR)—Medicina Aplicada, Departament de Medicina i Cirurgia, Universitat Rovira i Virgili (URV), Institut d’Investigació Pere Virgili (IISPV), Mallafré Guasch, 4, 43007 Tarragona, Spain; esther.guiu@urv.cat (E.G.-J.); tauguet.hj23.ics@gencat.cat (T.A.); alba.berlanga@urv.cat (A.B.); gemma.aragones@iispv.cat (G.A.); caguilar.hj23.ics@gencat.cat (C.A.); sandra.armengol@urv.cat (S.A.); 2 Servei Medicina Interna, Hospital Universitari Joan XXIII Tarragona, Mallafré Guasch, 4, 43007 Tarragona, Spain; aporras.hj23.ics@gencat.cat (J.A.P.); andreumano@gmail.com (A.M.); 3 Servei de Cirurgia, Hospital Sant Joan de Reus, Departament de Medicina i Cirurgia, Universitat Rovira i Virgili (URV), Institut d’Investigació Pere Virgili (IISPV), Avinguda Doctor Josep Laporte, 2, 43204 Reus, Spain; fatima.sabench@urv.cat (F.S.); mhernandezg@grupsagessa.com (M.H.); ddelcastillo@grupsagessa.com (D.C.); 4 Servei de Cirurgia, Hospital Universitari Joan XXIII Tarragona, Mallafré Guasch, 4, 43007 Tarragona, Spain; rjorba.hj23.ics@gencat.cat

**Keywords:** moderate obesity, fatty acid metabolism, adipose tissue, *de novo* fatty acid synthesis

## Abstract

The purpose of this work was to evaluate the expression of fatty acid metabolism-related genes in human adipose tissue from moderately obese women. We used qRT-PCR and Western Blot to analyze visceral (VAT) and subcutaneous (SAT) adipose tissue mRNA expression involved in *de novo* fatty acid synthesis (*ACC1*, *FAS*), fatty acid oxidation (*PPAR*α, *PPAR*δ) and inflammation (*IL6*, *TNF*α), in normal weight control women (BMI < 25 kg/m^2^, *n* = 35) and moderately obese women (BMI 30–38 kg/m^2^, *n* = 55). In SAT, *ACC1*, *FAS* and *PPAR*α mRNA expression were significantly decreased in moderately obese women compared to controls. The downregulation reported in SAT was more pronounced when BMI increased. In VAT, lipogenic-related genes and *PPAR*α were similar in both groups. Only *PPAR*δ gene expression was significantly increased in moderately obese women. As far as inflammation is concerned, *TNF*α and *IL6* were significantly increased in moderate obesity in both tissues. Our results indicate that there is a progressive downregulation in lipogenesis in SAT as BMI increases, which suggests that SAT decreases the synthesis of fatty acid *de novo* during the development of obesity, whereas in VAT lipogenesis remains active regardless of the degree of obesity.

## 1. Introduction

Obesity is significantly associated with the development of several comorbidities including type 2 diabetes mellitus, dyslipidemia, hypertension, metabolic syndrome, non-alcoholic fatty liver disease, cardiovascular disease and certain neoplasms [1]. Nevertheless, obesity itself does not necessarily lead to these comorbidities [2,3,4].

Not only fat accumulation in ectopic sites but also dysfunction of adipose tissue might play a significant role in defining an individual’s risk of developing obesity-related comorbidities [5]. Physiological and molecular studies have suggested that the fat stored in subcutaneous adipose depots are not directly implicated in the development of insulin resistance. It seems to have a “buffering” role due to the fact that it takes up fatty acids (FAs) and prevents other insulin-sensitive tissues from being exposed to their damaging consequences [6]. In this sense, Klein *et al.* showed that obesity-associated metabolic variables were not improved by liposuction (reduction of subcutaneous adipose tissue) [7]. However, reducing visceral adipose tissue by omentectomy combined with gastric banding has positive long-term effects on insulin sensitivity and glucose metabolism [8].

Likewise, deregulation of lipogenesis and FA oxidation contribute to the development of metabolic diseases [9]. The expression of *de novo* FA synthesis enzymes in human adipose tissue have been evaluated in some studies that have found lower mRNA expression in obese patients compared to control subjects [10,11,12,13,14,15]. With regard to FA oxidation, several reports have shown that activating the peroxisome proliferator-activated receptor aplha (PPARα) in human adipocytes enhanced FA oxidation by inducing the mRNA expression of the genes involved in this pathway [16,17]. Moreover, Wang *et al.* showed that targeted activation of peroxisome proliferator-activated receptor delta (PPARδ) in adipose tissue induces FA oxidation gene expression [18].

In a previous work, we studied the expression of the main genes involved in fatty acid metabolism in adipose tissue of morbidly obese and normal-weight control women [19]. Our findings suggested that, in morbid obesity, SAT prevents the subcutaneous fat mass from developing further. Because not all obese subjects have the same metabolic traits and the mechanisms of adipose tissue dysfunction are not fully understood, the aim of the present study was to use our previous findings to investigate whether the alterations in the fatty acid metabolism of morbidly obese women also manifest in moderately obese women. Consequently, we evaluated the expression of key genes related to *de novo* synthesis of FAs (*ACC1*, *FAS*), FA oxidation (*PPAR*δ, *PPAR*α) and inflammation (*IL6*, *TNF*α) in the SAT and VAT of moderately obese and normal-weight control women.

## 2. Results

### 2.1. Baseline Characteristics of the Cohort Studied

Subjects were classified according to BMI into control (BMI < 25 kg/m^2^), and moderately obese patients (BMI 30–38 kg/m^2^). The patients’ baseline characteristics are shown in Table 1. Moderately obese women had significantly higher levels of glucose metabolism variables (fasting glucose, insulin, HbA1c and HOMA2-IR) and triglycerides than the control group. HDL-C was significantly decreased in the moderately obese compared to controls.

Subsequently we sub-classified the moderately obese women according to the presence of diabetes. Obviously, the results indicate that glucose and HbA1c were significantly increased in diabetic patients (D) compared to non-diabetic subjects (ND) (Glucose: ND = 93.67 ± 2.73, D = 172.56 ± 21.58 mg/dL, *p* < 0.001; HbA1c: ND = 5.22 ± 1.83, D = 7.16 ± 1.83, *p* = 0.030).

**Table 1:** Characteristics of the cohort studied.

Variables	Controls (*n* = 35)	Moderately Obese Patients (*n* = 55)	*p-Value*
	Mean ± SD	Mean ± SD	
AGE (years)	49.57 ± 14.17	52.94 ± 14.24	0.289
WEIGHT (kg)	57.53 ± 7.17	84.49 ± 11.65	**<0.001**
WC (cm)	76.43 ± 11.42	109.85 ± 11.15	**<0.001**
BMI (kg/m^2^)	22.28 ± 1.63	33.67 ± 2.70	**<0.001**
GLUCOSE (mg/dL)	86.51 ± 22.96	110.61 ± 45.32	**0.002**
INSULIN (mU/L)	6.84 ± 5.43	13.88 ± 9.65	**<0.001**
HbA1c (%)	4.92 ± 0.64	5.49 ± 1.18	**0.015**
HOMA2-IR	0.88 ± 0.75	1.90 ± 1.38	**<0.001**
SBP (mmHg)	124.63 ± 18.20	130.37 ± 18.11	0.157
DBP (mmHg)	69.8 ± 9.52	74.27 ± 11.45	0.063
TOTAL CHOLESTEROL (mg/dL)	181.33 ± 37.57	182.92 ± 51.38	0.867
HDL-C (mg/dL)	55.34 ± 14.93	43.29 ± 10.86	**<0.001**
LDL-C (mg/dL)	107.33 ± 31.02	113.6 ± 43.37	0.482
TRIGLYCERIDES (mg/dL)	93.57 ± 58.13	149.15 ± 82.57	**0.001**

*p*-Values in bold indicate significant differences with respect to the control group (*p* < 0.05). BMI, body mass index; DBP, diastolic blood pressure; HbA1c, glycated haemoglobin; HDL-C, high-density lipoprotein; HOMA2-IR, homeostatic model assessment 2-insulin resistance; LDL-C, low-density lipoprotein; SBP, systolic blood pressure; WC, waist circumference.

### 2.2. Evaluation of the Expression of FA Metabolism Genes and Their Protein Levels in SAT and VAT

We performed the expression analysis of genes related to lipogenesis (*ACC1*, *FAS*), FA oxidation (*PPAR*α, *PPAR*δ) and inflammation (*IL6*, *TNF*α) in human adipose tissue.

**Figure 1:**
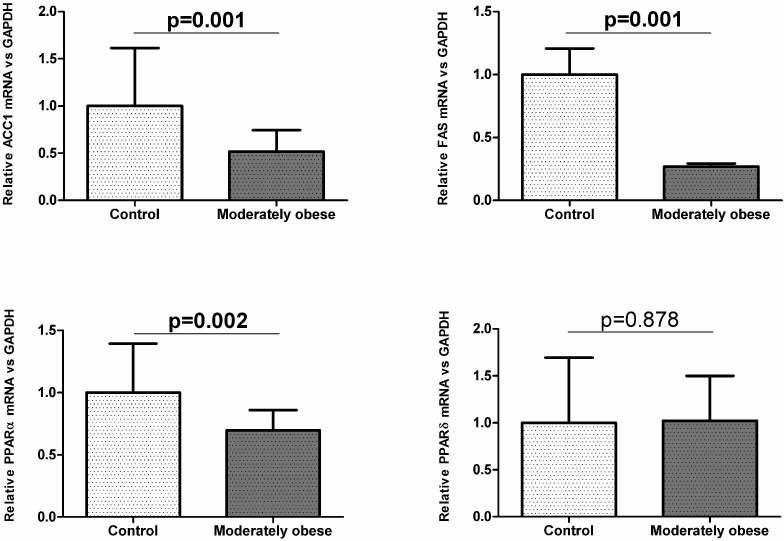

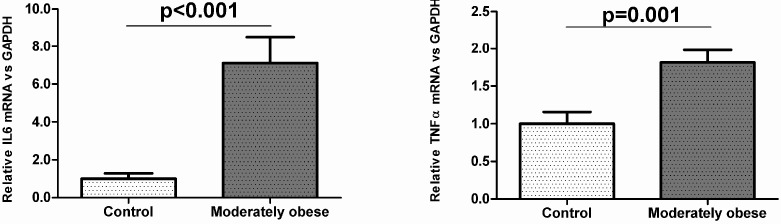
Expression of genes involved in lipogenesis, FA oxidation and inflammation in control (*n* = 35) and moderately obese women (*n* = 55) in subcutaneous adipose tissue. Student’s *t*-test was used to determinate differences between groups. Data are expressed as mean ± SD. The mRNA expression was calculated relative to the control group, whose mRNA expression was set to 1.0. ACC1, Acetyl-CoA carboxylase 1; FAS, Fatty acid synthase; IL6, Interleukin 6; PPARα, Peroxisome proliferator-activated receptor alpha; PPARδ, Peroxisome proliferator-activated receptor delta; TNFα, Tumor necrosis factor alpha.

**Figure 2:**
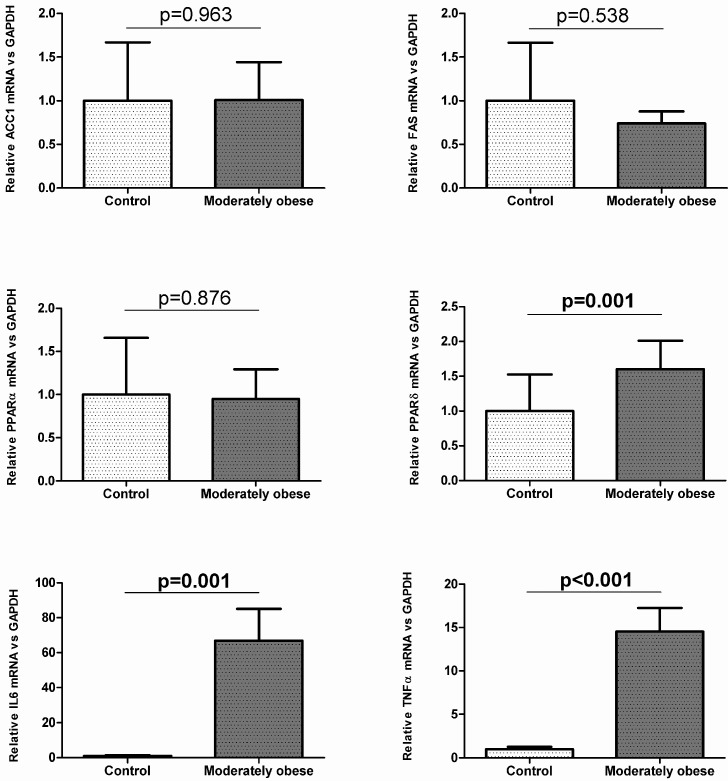
Expression of genes related to lipogenesis, FA oxidation and inflammation in control (*n* = 35) and moderately obese women (*n* = 55) in visceral adipose tissue. Student’s *t*-test was used to determinate differences between groups. Data are expressed as mean ± SD. The mRNA expression was calculated relative to the control group, whose mRNA expression was set to 1.0. ACC1, Acetyl-CoA carboxylase 1; FAS, Fatty acid synthase; IL6, Interleukin 6; PPARα, Peroxisome proliferator-activated receptor alpha; PPARδ, Peroxisome proliferator-activated receptor delta; TNFα, Tumor necrosis factor alpha.

**Figure 3:**
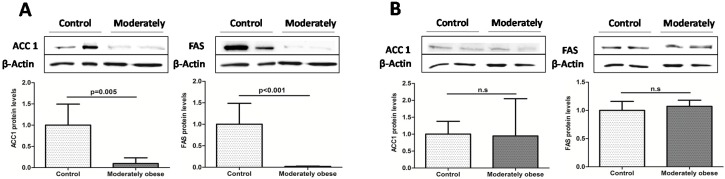
Western blot analysis of the main lipogenic enzymes in subcutaneous (**A**); and visceral (**B**) adipose tissue of moderately obese patients. Bar graphs show the quantification of ACC1 and FAS bands normalized by values of β-actin (*n* = 12 for each group). Student’s *t*-test was used to determinate differences between groups. Data are expressed as mean ± SD. ACC1, Acetyl-CoA carboxylase 1; FAS, Fatty acid synthase.

To investigate how gene expression was affected by BMI, we conducted analyses in the controls (BMI < 25 kg/m^2^) and moderately obese women (BMI 30–38 kg/m^2^). The subcutaneous mRNA expression of genes related to lipogenesis was significantly decreased in moderately obese women compared to control women (Figure 1). The results for VAT indicate that *ACC1* and *FAS* mRNA expression were similar in both groups (Figure 2). To validate these results, we also conducted Western Blot analysis of ACC1 and FAS in both fat depots. The protein analysis showed that ACC1 and FAS protein levels were similar to those obtained in the mRNA expression analysis. ACC1 and FAS protein levels in SAT were significantly lower in moderately obese patients (Figure 3A), whereas in VAT there were no differences between the two groups (Figure 3B).

In relation to the FA oxidation genes, our findings showed that visceral *PPAR*δ gene expression was significantly higher in moderately obese women than in controls (Figure 2), while subcutaneous mRNA expression was not significantly different in both groups (Figure 1). The subcutaneous mRNA expression of *PPAR*α was significantly lower in moderately obese women (Figure 1), whereas in VAT, *PPAR*α gene expression was similar in the two groups studied (Figure 2).

Regarding inflammation genes, the results showed that *IL6* and *TNF*α gene expression were significantly increased in the moderately obese women compared to control women in both tissues (Figure 1 and Figure 2).

Finally, the comparison of the mRNA expression of lipogenic and oxidative genes between moderately obese women and the morbidly obese women studied elsewhere [19] showed that the expression of the genes related to lipogenesis (*ACC1*, *FAS*) and FA oxidation (*PPAR*α, *PPAR*δ) was significantly lower in morbidly obese women than in moderately obese women in the SAT depot (*p* < 0.001). In the VAT depot, *ACC1* mRNA expression was downregulated in morbidly obese women (*p* = 0.013), while *FAS* gene expression was not significantly different in both types of obesity (*p* = 0.080). With regard to FA oxidation, *PPAR*δ mRNA expression was lower in morbidly obese women than in moderately obese women (*p* < 0.001), whereas *PPAR*α mRNA expression was similar in both groups (*p* = 0.124).

### 2.3. Correlation of VAT and SAT mRNA Expression with Parameters of Obesity and Glucose Metabolism

In VAT, we found that the oxidative gene *PPAR*δ correlated positively with BMI, weight and waist circumference (WC) (Table 2). *PPAR*α mRNA expression correlated negatively with HOMA2-IR (Table 2). On the contrary, *IL6* and *TNF*α gene expression correlated positively with HOMA2-IR, insulin, glucose and HbA1c (Table 2).

In SAT, we found that *FAS* mRNA expression has negative correlations with weight, BMI, WC, glucose, insulin and HOMA2-IR (Table 3). One of the key enzymes in lipogenesis, *ACC1*, correlated negatively with weight and BMI (Table 3). Also, our findings showed negative correlations between *PPAR*α expression and weight, BMI and WC (Table 3). Furthermore, *PPAR*δ correlated positively with HbA1c (Table 3). For the genes involved in inflammation, we found that *IL6* mRNA expression has positive correlations with weight, BMI, WC, glucose, insulin and HOMA2-IR (Table 3). *TNF*α gene expression also correlated positively with weight, BMI, WC, insulin and HOMA2-IR (Table 3).

**Table 2:** Correlations of visceral mRNA expression of genes involved in fatty acid metabolism and inflammation with anthropometric and glucose metabolism parameters.

Variables	*ACC1*		*FAS*		*PPAR*α		*PPAR*δ		*IL6*		*TNF*α	
	*r*	*p*-Value	*r*	*p*-Value	*r*	*p*-Value	*r*	*p*-Value	*r*	*p*-Value	*r*	*p*-Value
WEIGHT (kg)	−0.044	0.797	−0.010	0.957	−0.141	0.464	0.387	**0.018**	0.430	**0.020**	0.214	0.275
WC (cm)	0.196	0.521	0.291	0.335	−0.314	0.320	0.527	**0.044**	0.243	0.447	0.179	0.597
BMI (kg/m^2^)	−0.027	0.874	−0.044	0.812	−0.146	0.450	0.464	**0.004**	0.459	**0.012**	0.351	0.067
GLUCOSE (mg/dL)	−0.122	0.487	−0.153	0.411	−0.261	0.180	0.136	0.423	0.573	**0.001**	0.533	**0.004**
INSULIN (mU/L)	−0.045	0.808	−0.251	0.198	0.375	0.065	0.041	0.816	0.374	**0.046**	0.526	**0.007**
HbA1c (%)	0.105	0.587	−0.212	0.298	−0.031	0.887	0.221	0.233	0.496	**0.002**	0.427	**0.042**
HOMA2-IR	−0.087	0.642	−0.279	0.158	−0.418	**0.042**	0.062	0.730	0.479	**0.009**	0.558	**0.004**

BMI, body mass index; HbA1c, glycated haemoglobin; HOMA2-IR, homeostatic model assessment 2-insulin resistance; WC, waist circumference. The strength of association between variables was calculated using Pearson’s *r* correlation test. Bold numbers indicate statistically significant correlations (*p*-value < 0.05).

**Table 3:** Correlations of subcutaneous mRNA expression of genes involved in fatty acid metabolism and inflammation with anthropometric and glucose metabolism parameters.

Variables	*ACC1*		*FAS*		*PPAR*α		*PPAR*δ		*IL6*		*TNF*α	
	*r*	*p*-Value	*r*	*p*-Value	*r*	*p*-Value	*r*	*p*-Value	*r*	*p*-Value	*r*	*p*-Value
WEIGHT (kg)	−0.483	**<0.001**	−0.429	**<0.001**	−0.369	**0.003**	−0.002	0.990	0.488	**<0.001**	0.247	**0.030**
WC (cm)	−0.366	0.072	−0.388	**0.031**	−0.475	**0.026**	0.162	0.400	0.451	**0.018**	0.493	**0.005**
BMI (kg/m^2^)	−0.526	**<0.001**	−0.496	**<0.001**	−0.483	**<0.001**	0.000	0.998	0.449	**<0.001**	0.269	**0.018**
GLUCOSE (mg/dL)	−0.192	0.108	−0.240	**0.022**	−0.205	0.116	0.074	0.527	0.244	**0.040**	0.048	0.684
INSULIN (mU/L)	−0.216	0.081	−0.298	**0.013**	−0.180	0.185	0.182	0.132	0.275	**0.028**	0.264	**0.028**
HbA1c (%)	−0.042	0.750	−0.076	0.562	−0.127	0.367	0.264	**0.034**	0.107	0.428	0.147	0.257
HOMA2-IR	−0.216	0.082	−0.297	**0.013**	−0.183	0.177	0.188	0.120	0.341	**0.006**	0.238	**0.049**

BMI, body mass index; HbA1c, glycated haemoglobin; HOMA2-IR, homeostatic model assessment 2-insulin resistance; WC, waist circumference. The strength of association between variables was calculated using Pearson’s *r* correlation test. Bold numbers indicate statistically significant correlations (*p*-value < 0.05).

### 2.4. Relationship between the mRNA Expression of Genes Involved in Lipogenesis, FA Oxidation and Inflammation in VAT and SAT

The associations between the expression of the genes involved in lipogenesis point out that *FAS* mRNA expression was directly related to *ACC1* mRNA expression in both VAT and SAT depots (SAT: *r* = 0.822, *p* < 0.001; VAT: *r* = 0.417, *p* = 0.018). In regard to FA oxidation correlation analysis, the findings showed that *PPAR*α and *PPAR*δ mRNA expression were not related in both fat tissues. Finally, *IL6* gene expression had a positive correlation with *TNF*α gene expression in both tissues (SAT: *r* = 0.314, *p* = 0.004; VAT: *r* = 0.342, *p* = 0.05).

## 3. Discussion

In the present work, we investigated the expression of crucial genes in fatty acid metabolism in VAT and SAT paired samples from moderately obese and normal-weight women. Although some studies of fatty acid metabolism in human adipose tissue have been published [10,12,13,14,20,21], the originality of the present work resides in the fact that it provides a validated study of fatty acid metabolism in both adipose tissues simultaneously in moderate obesity.

The main findings of this work show that the gene expression of the main enzymes related to *de novo* fatty acid synthesis (*ACC1*, *FAS*) and *PPAR*α was similar in the two groups in VAT, but different in SAT. Their subcutaneous mRNA expression was significantly downregulated in moderately obese women.

It should also be noted that when the mRNA expression of these genes in moderately obese women was compared to the expression in morbidly obese women studied elsewhere [19], we found that the subcutaneous mRNA expression of all the genes studied was lower in morbidly obese women; that is to say, mRNA expression decreases when BMI increases.

In our study, the lipogenic pathway is, at mRNA and protein expression levels, downregulated in the subcutaneous fat depot of moderately obese women. Although an increase of *de novo* FA synthesis is expected in the development of obesity, our findings agree with those of other authors [22,23]. They indicate that the downregulation of the lipogenesis pathway in the obese cohort is a late and adaptive process that prevents the fat mass from developing further. In this sense, mice lacking the lipogenic enzyme FAS in adipose tissue manifested resistance to diet-induced obesity and increased energy expenditure. Also, Lodhi *et al.* found a decreased adipogenesis activity in *FAS* knockdown embryonic fibroblasts [24].

As far as the expression of genes related to FA oxidation was concerned, our results showed that in SAT *PPAR*δ gene expression was similar in the two groups studied, whereas in VAT it was upregulated in moderately obese women. In SAT *PPAR*α gene expression was downregulated, while in VAT it was similar in the two groups. The decreased FA oxidation in SAT in moderately obese women might be explained, at least in part, because mitochondrial function-related genes are downregulated in male and female obese subjects [25]. Moreover, in morbidly obese patients, MacLaren *et al.* found an increase in lipid storage and lipolytic genes, but a decrease in *de novo* triglyceride synthesis and oxidative genes in SAT [26]. The role of these PPARs in white adipose tissue in the pathophysiology of obesity has yet to be elucidated. New prospective studies are needed to clarify their function in obese adipocytes.

Besides the processes described above, there are also others involved in fat accumulation in white adipose tissue such as FA uptake or lipolysis. It is important to note, that all these processes, occur at different rates and amounts in obese and normal-weight individuals, depending upon the anatomical location of the adipose tissue, and also according to gender and grade of obesity [27].

It is well known that the increased IL6 and TNFα circulating levels in obese patients have led to the conclusion that obesity is characterized by a subjacent chronic low-grade inflammation [28]. In this sense, our results showed increased *IL6* and *TNF*α gene expression in moderately obese women in comparison with the control group in both tissues.

Our results reinforce the hypothesis that SAT, from a metabolic point of view, is less harmful than VAT [29]. Recent studies have analyzed whether subcutaneous, intra-abdominal and hepatic fat were related to insulin resistance and lipidic parameters. Only subcutaneous fat was not significantly correlated to these variables [30]. *In vitro* and *in vivo* studies of the physiology of adipose tissue confirm that lipolysis and fatty acid uptake rates are not the same in SAT as in VAT. SAT appears to be more passive than VAT and to limit the detrimental effects of ectopic fat deposition by the long-term accumulation of excess FAs [6]. Also, subcutaneous fat is related to a favorable adipokine profile [6]. In this respect, individuals with Cushing’s syndrome or congenital lipodystrophies tend to have increased metabolic and cardiovascular risk despite having a marked reduction in subcutaneous fat [31,32]. Moreover, several reports have shown that regional subcutaneous fat mass is inversely associated with fasting insulin levels and insulin levels after an oral glucose load, and positively associated with insulin sensitivity [33,34,35,36]. In agreement with these results, we found that FAS mRNA expression is inversely associated with insulin, glucose and HOMA2-IR. Data on the beneficial metabolic consequences of SAT, the deleterious effects of its deficiency [37,38] and the positive effects of its transplantation into VAT depots in mice [39] suggests that SAT plays a “buffering” role in obesity due to the fact that it prevents excess supply of lipids from spilling over into “ectopic” sites [40].

Our study cohort allowed us to investigate lipogenic and FA oxidation pathways in SAT and VAT fat depots without the interference of confounding factors like gender or age. Only women were included because it is well known that men and women differ substantially in regard to body composition, energy imbalance, sex hormones and adipokines [41,42]. Moreover, several studies showed sex-specific differences in lipid and glucose metabolism [43]. We were also able to extrapolate the results found in the morbidly obese cohort [19] to the moderately obese cohort. Nevertheless, the results of our study cannot be extrapolated to men.

## 4. Material and Methods

### 4.1. Subjects

The study was approved by the ethics committee of the Hospital Sant Joan de Reus and all subjects gave written informed consent before taking part in the study. The majority of the patients in the Hospital Sant Joan de Reus who undergo bariatric surgery or laparoscopic cholecystectomy are women. Therefore, adipose tissue samples were from 105 Caucasian women. Of these, 55 were moderately obese (body mass index (BMI) 30–38 kg/m^2^) and 35 were normal-weight controls (BMI < 25 kg/m^2^). SAT and VAT samples were obtained from moderately obese patients who had undergone bariatric surgery (patients with BMI ≥ 37 kg/m^2^) and laparoscopic cholecystectomy for benign gall bladder disease or laparoscopic hiatus hernia repair (patients with BMI < 37 kg/m^2^) and from normal-weight subjects who had undergone laparoscopic abdominal surgery (described in detail elsewhere) [19].

The moderately obese and normal-weight women were of similar ages. The body weight of the moderately obese group had not fluctuated be more than 2% for at least three months before surgery. The exclusion criteria were: (1) patients who were taken antidiabetic or hypolipemiant drugs; (2) diabetic women receiving insulin; (3) subjects undergoing contraceptive treatment; (4) patients who had an acute illness, inflammatory or infectious diseases or neoplastic malignant diseases.

Of the moderately obese women, 16% were diagnosed with type 2 diabetes mellitus based on ADA guidelines [44]. These patients were following a dietetic treatment. All the usual exclusion criteria were taken into account.

### 4.2. Biochemical Analyses

Each of our patients was evaluated with a complete physical, anthropometrical and biochemical assessment. Total cholesterol, HDL-C, LDL-C, triglycerides, glucose, insulin and HbA1c were measured using a conventional automated analyzer after overnight fasting. Insulin resistance (IR) was calculated using HOMA2-IR [45].

### 4.3. RNA Isolation and Gene Expression

Total RNA was extracted from SAT and VAT by using the RNeasy mini kit (Qiagen, Barcelona, Spain) and was reverse transcribed to cDNA using the High Capacity RNA-to-cDNA Kit (Applied Biosystems, Madrid, Spain). Real-time quantitative PCR was carried out with TaqMan Assay predesigned by Applied Biosystems for the detection of *ACC1*, *FAS*, *PPAR*α, *PPAR*δ, *IL6*, *TNF*α and GAPDH gene. All reactions were performed in duplicate using the 7900HT Fast Real-Time PCR systems. SAT and VAT mRNA expression of the genes mentioned above was calculated relative to the mRNA expression of Glyceraldehyde-3-Phosphate Dehydrogenase (GAPDH).

### 4.4. Western Analysis

Protein levels of ACC1 and FAS were assayed by Western Blot. Frozen SAT and VAT tissue samples from 24 individuals (MO, *n* = 12; Control, *n* = 12) were homogenized in lysis buffer (50 mM HEPES, 150 mM NaCl, 1 mM EDTA, 1 mM DTT, 0.1% SDS, 100 mM NaF, 30 mM Na_4_O_7_P_2_ and 1% protease inhibitor cocktail (Thermo Scientific, Madrid, Spain)). Protein concentration was determined using BCA assay kit (Thermo Scientific). Samples were separated by SDS/PAGE and transferred electrophoretically to nylon membranes. Membranes were blocked by incubation in a solution of 5% skimmed milk and were probed using antibodies against ACC1, FAS and β-actin (Cell Signaling, Danvers, MA, USA). Anti-rabbit IgG or anti-mouse IgG (Thermo Scientific) were used as secondary antibody. Immunodetection of the protein was done using SuperSignal West Pico or Femto Chemiluminescent kit (Thermo Scientific). Finally, band densitometry was analyzed using Phoretix1D software.

### 4.5. Statistical Analyses

Results are expressed as mean ± SD (standard deviation). Student’s *t-*test or one-way ANOVA were carried out to determinate differences between groups. Univariate association was tested by Pearson (parametric variables) or Spearman (nonparametric variables) correlation analysis. We used SPSS/PC+ statistical package (version 22.0; SPSS, Chicago, IL, USA) for the statistical analyses. *p*-values < 0.05 were considered statistically significant.

## 5. Conclusions

The results reported here suggest that, in moderate obesity, subcutaneous fat has a defense mechanism against an excess of fatty acid accumulation by diminishing the expression of lipogenic-related genes, while visceral fat does not. Interestingly, the extrapolation of the results found in the morbidly obese cohort [19] to the moderately obese cohort showed that this downregulation reported in subcutaneous adipose tissue increases as BMI increases. As far as FA oxidation is concerned, future studies are necessary to gain further knowledge about PPARs regulation in white adipose tissue of obese subjects.

## Abbreviations

ACC1, acetyl-coenzyme A carboxylase 1; BMI, body mass index; DM2, diabetes mellitus type 2; FA, fatty acid; FAS, fatty acid synthase; GAPDH, glyceraldehyde-3-phosphate dehydrogenase; HbA1c, glycated haemoglobin; HDL-C, high density lipoprotein cholesterol; HOMA2-IR, homeostatic model assessment method insulin resistance; IL6, interleukin 6; IR, insulin resistance; LDL-C, low density lipoprotein cholesterol; PPARα, peroxisome proliferator-activated receptor α; PPARδ, peroxisome proliferator-activated receptor δ; PPARγ, peroxisome proliferator-activated receptor γ; SAT, subcutaneous adipose tissue, TNFα, tumor necrosis factor; VAT, visceral adipose tissue; WC, waist circumference.
